# A rapid and robust assay for detection of S-phase cell cycle progression in plant cells and tissues by using ethynyl deoxyuridine

**DOI:** 10.1186/1746-4811-6-5

**Published:** 2010-01-28

**Authors:** Edit Kotogány, Dénes Dudits, Gábor V Horváth, Ferhan Ayaydin

**Affiliations:** 1 Cellular Imaging Laboratory, Biological Research Center, Hungarian Academy of Sciences, Temesvári krt 62, 6726 Szeged, Hungary; 2 Institute of Plant Biology, Biological Research Center, Hungarian Academy of Sciences, Temesvári krt 62, 6726 Szeged, Hungary

## Abstract

**Background:**

Progress in plant cell cycle research is highly dependent on reliable methods for detection of cells replicating DNA. Frequency of S-phase cells (cells in DNA synthesis phase) is a basic parameter in studies on the control of cell division cycle and the developmental events of plant cells. Here we extend the microscopy and flow cytometry applications of the recently developed EdU (5-ethynyl-2'-deoxyuridine)-based S-phase assay to various plant species and tissues. We demonstrate that the presented protocols insure the improved preservation of cell and tissue structure and allow significant reduction in assay duration. In comparison with the frequently used detection of bromodeoxyuridine (BrdU) and tritiated-thymidine incorporation, this new methodology offers several advantages as we discuss here.

**Results:**

Applications of EdU-based S-phase assay in microscopy and flow cytometry are presented by using cultured cells of alfalfa, Arabidopsis, grape, maize, rice and tobacco. We present the advantages of EdU assay as compared to BrdU-based replication assay and demonstrate that EdU assay -which does not require plant cell wall digestion or DNA denaturation steps, offers reduced assay duration and better preservation of cellular, nuclear and chromosomal morphologies. We have also shown that fast and efficient EdU assay can also be an efficient tool for dual parameter flow cytometry analysis and for quantitative assessment of replication in thick root samples of rice.

**Conclusions:**

In plant cell cycle studies, EdU-based S-phase detection offers a superior alternative to the existing S-phase assays. EdU method is reliable, versatile, fast, simple and non-radioactive and it can be readily applied to many different plant systems.

## Background

Detection of cell proliferation is a fundamental method for assessing cell health, determining genotoxicity, and evaluating stress responses. The most accurate method utilizes direct measurement of new DNA synthesis. Traditionally, this has been performed by incorporating tritium-labeled thymidine and detection by autoradiography [1]. Because of the involvement of radioactivity, this method has been replaced by incorporation of a thymidine analog such as bromodeoxyuridine (BrdU) into DNA, followed by immunodetection with a specific antibody raised against the thymidine analog [2]. Although being effective, this method requires DNA denaturation or digestion (using hydrochloric acid, heat or DNase) to expose BrdU to the antibody. This step is lengthy, difficult to perform consistently, and can adversely affect the morphology of the sample. Antibody-based detection method of BrdU assay also necessitates cell wall digestion in experiments carried out on plant cells. Therefore protoplasts, partially cell-wall-digested cells and organs or tissue sections are often used for BrdU-based detection of proliferative activity in plants [3].

However, treatment with cell wall digesting enzymes imposes a significant wounding and osmotic stress on plant cells. Moreover, types and concentrations of the enzymes and the osmolarity of the digestion medium should also be specifically optimized for each plant species, organ and cell type under investigation [4]. Partial cell wall digestion or release of protoplasts not only prolong the experimental duration but also cause substantial reorganization of cytoskeleton and activation of stress and defense-related genes. To alleviate the stress-related artifacts, it is also possible to first chemically fix the cells and then partially digest cell walls. However, this approach requires highly pure and expensive cell wall digestion enzymes, as crude enzyme preparations contain impurities such as proteases and nucleases that can significantly compromise cellular integrity [5].

EdU (5-ethynyl-2'-deoxyuridine) is a terminal alkyne-containing nucleoside analog of thymidine, and is incorporated into DNA during active DNA synthesis [6]. EdU detection method is based on click chemistry [7]. In a Cu(I)-catalyzed reaction, the alkyne of EdU reacts with an azide containing fluorochrome, forming a stable covalent bond. EdU-based assay has been successfully used in detection of proliferation in avian cochlea [8], in chick embryos [9] in breast cancer cells [10] and in human fibroblasts [11]. Twenty-four hours long EdU incubation duration has been recently used in Arabidopsis root tips to identify dysfunction of the quiescent center [12], but the possibility of very short EdU pulse labeling to determine S-phase indices, the suitability of this novel detection method in various plant species, comparison of EdU assay to BrdU assay and plant specific parameters and fields of application such as plant flow cytometry have not been explored in detail.

Here we show the advantages in microscopy and flow cytometry applications of this novel S-phase detection assay using various cultured plant cells and root meristems.

## Results and Discussion

### EdU-based assay versus immunodetection of BrdU

**Figure 1:**
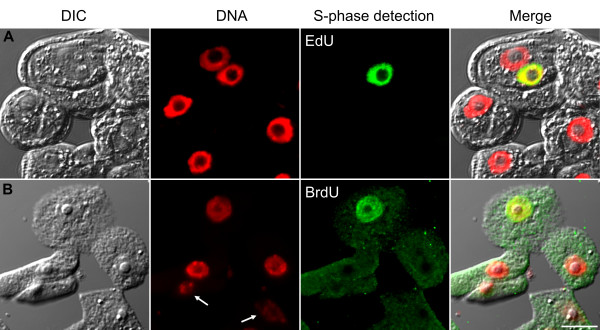
**Comparison of EdU-based and BrdU-based replication assays on Arabidopsis suspension culture**. **(A) **EdU click reaction-based replication assay. Mild detection conditions without cell wall digestion or DNA digestion led to a better preserved cellular and nuclear morphology. Following 2 h 10 μM EdU incubation on 2d-old Arabidopsis culture, incorporated EdU was detected with click reaction using Alexa Fluor 488 azide (green). **(B) **BrdU-based replication assay on 2d-old Arabidopsis culture following 2 h 10 μM BrdU incubation. Harsh detection conditions resulted in partial loss of cellular and nuclear integrity. Alexa Fluor 488 antibody was used for immunodetection of BrdU (green). Nuclei were counterstained with DAPI and pseudocolored red. Arrows indicate nuclei with weak DNA staining. Differential interference contrast (DIC) transmission images were overlaid onto fluorescence images at the last panel. Scalebar: 10 μm.

To compare EdU assay with BrdU immunodetection, *Arabidopsis thaliana *sp. Columbia suspension culture cells were incubated with 10 μM EdU for 2 hrs and fixed with detergent-containing formaldehyde. Since EdU detection does not rely on antibodies and the molecular size of the chemicals used during detection is small compared to the size of the antibody molecules, cells were directly incubated with the detection cocktail without performing cell wall digestion. The detection cocktail is composed of an azide-conjugated fluorochrome (such as Alexa Fluor 488) and copper (I) as the catalyzer of the click reaction [13]. Stained nuclei with high signal-to-noise ratio were easily detectable under the microscope (Figure 1A). Omission of cell wall digestion and DNA denaturation/digestion steps resulted in better preservation of cellular structures judged by DAPI (4',6-diamidino-2-phenylindole) staining of the nuclei and by differential interference contrast (DIC) microscopy (Figure 1A). As comparison, 10 μM (2 hrs) BrdU-labeled and similarly fixed Arabidopsis cells were processed for immunodetection of BrdU. Following fixation washes, cell walls were partially digested with an enzyme cocktail composed of high purity cellulase and pectinase. Cells were settled on poly-L-lysine coated slides and plasma membranes were permeabilized with Triton X-100 followed by DNase I digestion. Anti-BrdU antibody incubation was followed by fluorochrome-conjugated secondary antibody incubation. Similar to EdU-labeled nuclei, BrdU-labeled nuclei were also detectable albeit with high background noise (Figure 1B). Cell wall digestion and DNase treatment often resulted in partial loss of globular cell shape and barely detectable nuclei with a melted look (Figure 1B arrows). On the other hand, EdU assay resulted in morphologically well-preserved cells with clearly identifiable nuclei brightly stained with the DNA dye DAPI (Figure 1A). Clear determination of the total number of nuclei following EdU assay was particularly advantageous for correct assessment of S-phase index. Moreover, total experimental duration of EdU assay was calculated to be 6 hours shorter than that of BrdU immunolabeling procedure (Table 1). On the other hand, one unique advantage of immunodetection-based assay is that, availability of specific antibodies against halogenated thymidine analogs such as, chlorodeoxyuridine, iododeoxyuridine as well as bromodeoxyuridine allows multiplexing experiments to analyze replication initiation and temporal dissection of S-phase [14]. Sharpless and coworkers reported the ruthenium-catalyzed cycloaddition of azides to alkynes [15]. While the Cu(I)-catalyzed EdU-based click reaction is limited to terminal alkynes, the Ru(II)-catalyzed reaction is active with internal alkynes, as well. Therefore, similar multiplexing experiments may also be possible with the future development of EdU-analogous chemicals that have internal alkyne groups.

**Table 1:** Comparison of experimental steps and durations for EdU-assay and BrdU-based immunolocalization

	EdU assay	BrdU immunolocalization
EdU or BrdU labeling	2 h*	2 h*

Fixation and washes	15 min + 15 min	15 min + 15 min

Cell wall digestion and washes	-	30 min + 15 min

Plasma membrane permeabilization and washes	-	30 min + 15 min

DNase I digestion and washes	-	30 min + 15 min

EdU click reaction or BrdU antibody incubations	30 min	3 h + 25 min + 1 h

Washing with DAPI and sample mounting	20 min	20 min

**Total experimental duration**	**3 h 20 min**	**9 h 30 min**

* Depending on the proliferation activity of a given cell type, shorter incubations may be sufficient or longer incubations may be necessary.

### EdU assay is a versatile method for both monocot and dicot plant species

**Figure 2:**
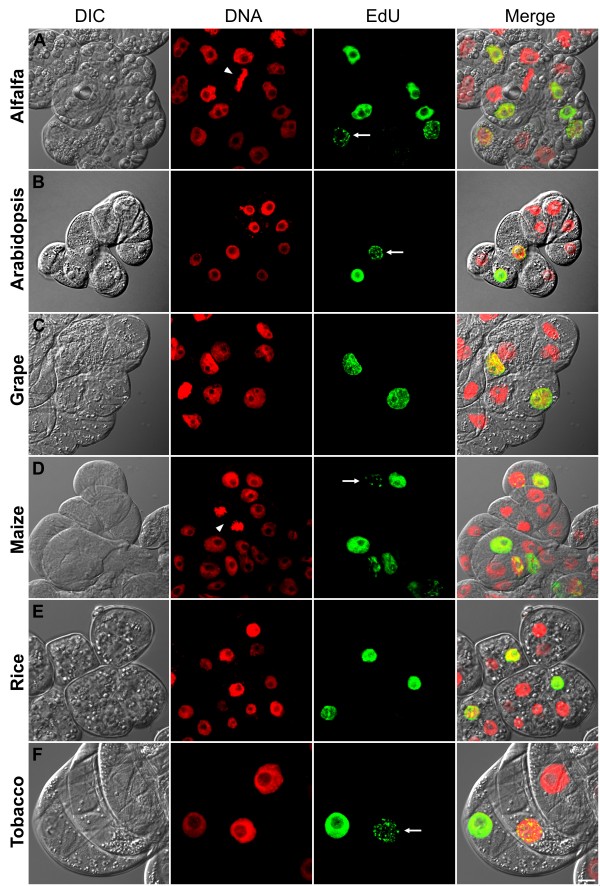
**EdU-based replication assay on 2d-old monocot and dicot plant cultures**. EdU (10 μM, 2 h) incorporated cells were detected with click reaction using Alexa Fluor 488 azide (green panel). DNA is counterstained with DAPI (red). Transmission images (DIC) were overlaid onto fluorescence images at the last panel. **(A) **Alfalfa (*M. sativa *ssp. varia A2), **(B) **Arabidopsis (*A. thaliana *ecotype Columbia), **(C) **Grape (*V. berlandieri *× *V. rupestris *cv. 'Richter 110'), **(D) **Maize (*Z. mays*, cv. H1233), **(E) **Rice (*O. sativa *ssp. japonica cv. 'Unggi 9'), **(F) **Tobacco (*N. tabacum *cv. Petit Havana SR1). Arrows in green panel indicate spotty labeled early S-phase cells. Arrowheads in red panel show cells in mitosis. Scalebar: 10 μm.

**Figure 3:**
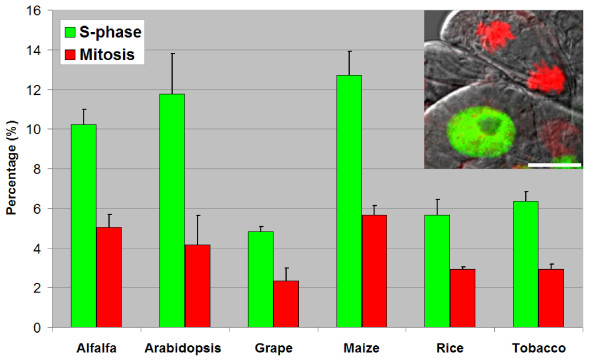
**Ratio of EdU-labeled and mitotic cells as dual parameter proliferation analysis of 2d-old plant cultures**. EdU-labeled S-phase cells (in addition to cells which were recently in S-phase) and mitotic cells were scored as frequencies for the indicated cultures using EdU assay (green) and DAPI labeling (red), respectively. EdU was used at 10 μM concentration for 2 h. Inset photo shows an EdU-labeled maize cell (green) next to a cell in telophase stage of mitosis (red). In two independent experiments, more than 500 cells were counted for each culture. Scalebar: 10 μm.

**Figure 4:**
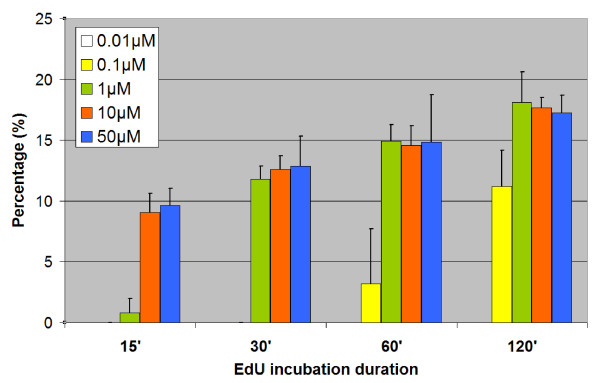
**The effect of EdU concentration and incubation duration on EdU labeling index of 36 h-old Arabidopsis cultures**. EdU-Alexa Fluor 488-labeled cells were scored as frequencies for the indicated EdU concentrations and incubation durations following EdU assay on 36 h-old Arabidopsis cultures. In two independent experiments, more than 500 cells were counted for each experimental condition.

The thickness as well as the composition and organization of plant cell walls can vary significantly between species and also differ between cell types and developmental stages [16]. Therefore, we have tested EdU assay on various monocot and dicot plant suspension cultures. Two days after subculturing, alfalfa, Arabidopsis, grape, maize, rice and tobacco cells were treated for 2 hours with 10 μM EdU, fixed and incubated with EdU detection cocktail. Nuclei were counterstained with DAPI and cells were analyzed by confocal laser scanning microscope (Figure 2). EdU-labeled nuclei were clearly detected in all cultured cells tested. While cells that were just entered into S-phase after the addition of EdU showed complete labeling of the nuclei, those cells which were entered into replication phase near to the end of the 2 hours labeling period showed spotty labeling (Figure 2A, B, D, F, arrows in green panel). These patchy-labeled early S-phase nuclei were well preserved due to absence of harsh treatments during the detection protocol. Mild reaction conditions were also favorable from the point of superior preservation of chromosomal morphology in mitotic cells (Figure 2A, D, arrowheads and Figure 3 inset). This was particularly helpful to assess proliferation both by S-phase index and by mitotic index. Dual parameter analysis of the cell cycle for the tested cultures indicated that maize culture has the highest number of S-phase cells followed by Arabidopsis and alfalfa cultures after two days of subculturing (Figure 3). Relatively low DNA synthetic activity was detected for tobacco (SR1) and rice (Unggi 9) cultures after 2 hrs EdU incubation. Lowest frequency of S-phase cells was detected in grape culture, which can be explained by the recent initiation of this cell line (Figure 3). The differences in proliferation activity among the cultures may be explained by differences in cell cycle durations and/or differential adaptation to liquid culture and hormone conditions. To determine the relationship between EdU concentration and labeling duration, we have treated Arabidopsis cultures with various EdU concentrations for different labeling durations (Figure 4). Incubation durations as short as 15 minutes were possible using 10 μM or 50 μM EdU concentrations for the fast growing Arabidopsis culture after 36 hrs of subculturing. For 1 μM EdU concentration, at least 30 minutes incubation was necessary to obtain easily detectable signal intensity. Using less than 1 μM EdU concentration was not sufficient for S-phase index determination due to extremely low fluorescence intensities of the labeled nuclei (Figure 4, 0.1 μM and 0.01 μM). Based on the values of Figure 3 (10 μM EdU, 2 hrs labeling), we predict that for slow growing cultured cells having 2-3 times less EdU-labeling index than that of Arabidopsis cells, 30 to 60 minutes-long incubation with at least 10 μM EdU concentration may be needed as minimum labeling duration. On the other hand, longer incubations can also be used to assess the frequency of cells, which entered and exited S-phase during the labeling period. Due to its cumulative effect, this technique can be especially useful to determine fine differences in S-phase regulation as well as to assess the length of G2 phase by determining minimum incubation duration needed for the first appearance of EdU-labeled mitotic chromosomes. Nevertheless, simple and versatile EdU assay can be used to rapidly screen the proliferation status of several *in vitro *plant cultures as well as to assess proliferation response after various treatments or genetic modifications.

### Using EdU assay in flow cytometry of plant nuclei

**Figure 5:**
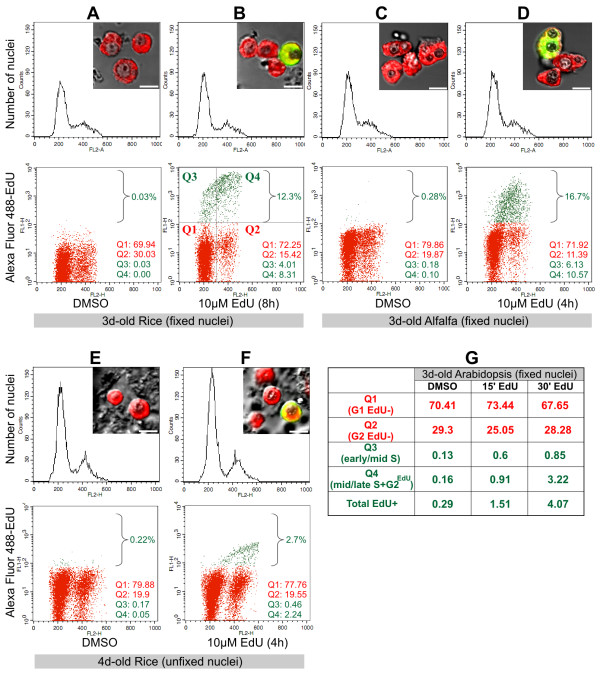
**Cell cycle analysis with EdU using flow cytometry**. Formaldehyde fixed **(A-D **and **G) **or unfixed **(E, F) **nuclei isolated from EdU-treated and untreated rice (**A, B, E, F**), alfalfa (**C, D**) and Arabidopsis (**G**) cultures were analyzed by flow cytometry. Uniparametric DNA histograms of propidium iodide intensity (FL2-A) are shown at upper panels. Lower panels show biparametric dot-plot analysis of Alexa Fluor 488-EdU intensity (FL1-H, logarithmic scale) versus propidium iodide intensity (FL2-H). Braces in biparametric plots indicate the total EdU-labeled population or background green fluorescence above EdU threshold (see Methods). Inset photos show fluorescence images of isolated nuclei counterstained with DAPI (red) following EdU assay with Alexa Fluor 488 azide (green). Scalebars are 5 μm. An example of quadrant thresholds is shown for biparametric plot of Figure 5B. The ratios of nuclei in each quadrant are displayed as percentage on the lower right corners of each biparametric plot. **(G) **Ratio of indicated biparametric fractions (quadrants Q1-Q4) after EdU (15' and 30') and DMSO treatment of Arabidopsis suspension cultures.

Cell-cycle-phase distribution including DNA synthesis activity of a given *in vitro *culture or *in vivo *organ can effectively be determined by flow cytometry after isolation of nuclei. In animal cell cultures, high quality and intact nuclei preparations can be relatively easily obtained in hypotonic buffers with detergents. In plants, however, cell walls pose a significant barrier for nuclei isolation. To alleviate this problem, cell walls can be removed by protoplast isolation followed by mechanical disruption in hypotonic buffers with detergents. However, removal of cell walls can require hours during which period cells cease entering into mitosis or DNA replication phase due to severe wounding stress and osmotic shock. Therefore, this method is not suitable for determining kinetic parameters of cell division cycle. As an alternative, Galbraith *et al*. [17] used a practical approach in which the suspensionsof intact nuclei are prepared by chopping a small amount offresh tissue in a suitable isolation buffer. This method affords high quality DNA histograms and provides immediate release of nuclei from cells or tissues and is currently widely used for flow cytometry of unfixed nuclear samples of plant cells [18,19]. Formaldehyde fixation of samples offers convenience in experiments involving multiple samples and timepoints such as cell cycle synchronization. However, fixed nuclear preparations often display wider G1 and G2 peaks in flow cytometry histograms making uniparametric S-phase estimations difficult. As an alternative to single parameter flow cytometry analysis, BrdU immunodetection combined with DNA staining can be used as biparametric (bivariate or dual-parameter) analysis of fixed nuclei for reliable determination of various cell cycle phases and cell cycle dynamics without using estimation algorithms [20]. Similar to microscopy-based immunodetection of BrdU, detection of BrdU in flow cytometry also requires DNA denaturation before the use of antibodies. Since excessive DNA denaturation adversely affects the quality of the nuclei, the concentration, duration and temperature of the digestion or denaturation process need to be meticulously optimized [20,21]. Moreover, treatment with antibodies prolongs the overall experimental duration and adds an extra step of complexity. Due to its simplicity, EdU-based method can be a superior alternative to BrdU-based flow cytometry especially in plant cell studies. EdU-based biparametric flow cytometry has recently been reported in breast cancer cells [10] and its efficiency has been demonstrated as compared to BrdU-based flow cytometry assay in human leukemia cells [13], however, its use in plant cell flow cytometry, to our knowledge, has not been reported, yet. We have used a monocot (rice) and two dicot (alfalfa, Arabidopsis) suspension cultures for biparametric flow cytometry analysis (Figure 5). After 10 μM EdU (or 0.1% DMSO) treatment, cultures were directly chopped with a razor blade in nuclei isolation buffer to release nuclei. Formaldehyde fixed (Figure 5A-D and 5G) or unfixed (Figure 5E, F) samples were incubated in EdU detection cocktail followed by DNA counterstaining with DAPI (for microscopy) or with propidium iodide (for flow cytometry). Using an aliquot of these samples, EdU-labeled nuclei were checked for quality of labeling as well as integrity of nuclei by DAPI staining. (Figure 5 inset photos). Remaining nuclei preparations were analyzed by uniparametric (DNA content by propidium iodide, Figure 5 upper panels) and by biparametric flow cytometry (Alexa Fluor 488-EdU intensity versus DNA content, Figure 5 lower panels). After 3 days of subculturing, rice cells treated with EdU for eight hours displayed 12.3% EdU labeled nuclei as a clearly separated cluster in bivariate dot-plot (Figure 5B). Based on their DNA content, the nuclei of this cluster represent the cells that were in early, mid and late S-phase in addition to the cells that already entered into G2 phase during the labeling duration. For faster growing alfalfa cells (3d-old) which were incubated for 4 hours with EdU, this cluster displayed nearly 17% EdU-labeled nuclei (Figure 5D). To determine nuclear autofluorescence and non-specific binding of Alexa Fluor 488 azide, control cultures were incubated with 0.1% DMSO (solvent of EdU) and similarly processed as in EdU-treated samples. Less than 0.3% of the total nuclei were detected above the "EdU threshold" value (see Methods) indicating the specificity of the assay (Figure 5A, C, E and DMSO in 5G). Unlike antibody-based BrdU assay, click reaction should not be sensitive to cellular proteases released during chopping procedure; therefore, we have tested the suitability of EdU assay (10 μM EdU, 4 h) on unfixed nuclei of 4d-old rice cultures. Fresh nuclear preparations should be kept cold to prevent degradation of samples. While EdU click reaction was not efficient on ice-cold temperatures, after 30 minutes incubation at room temperature, we could successfully detect EdU incorporated nuclei both by flow cytometry and by microscopy (Figure 5F). Unfixed nuclei displayed significantly better monoparametric histograms (Figure 5E, F, upper panels) with narrower G1 and G2 peaks. On the other hand, on several independent experiments, we have observed noticeably higher signal intensities of EdU-labeled fixed nuclei as compared to unfixed nuclei. Nevertheless, from chopping of cells to flow cytometry analysis, the total duration of biparametric EdU assay was considerably shorter than BrdU assay using either fixed (1.5 hrs) or unfixed (45 mins) nuclei (see Methods). To assess the minimum feasible EdU incubation duration, we have tested 15 and 30 minutes of EdU incubations (10 μM) on 3-days-old Arabidopsis cultures. Incubation durations as short as 15 and 30 minutes were enough to obtain labeling indices higher than DMSO treated control nuclei (Figure 5G, Total EdU+).

Based on these experiments, the advantages of EdU assay for use in plant flow cytometry were readily evident. Clearly defined EdU-labeled population on a logarithmic scale in biparametric plots allowed us to precisely determine the fraction of cells labeled with EdU, hence giving information on the dynamics of S-phase entry and progression of cells into G2 phase of the cell cycle. Especially with short EdU pulse durations, dual parameter analysis with EdU is very informative and advantageous as subpopulations of S-phase (early, mid and late S-phase) can be better assessed. As an example, Figure 5B displays a simple quadrant analysis. Q1 and Q2 sectors indicate the population of 2C and 4C nuclei that never entered into replication phase during EdU labeling period. Q3 fraction represents nuclei entered into S-phase relatively recently. Q4 fraction indicates nuclei entered into S-phase earlier than that of Q3 fraction during the EdU labeling period. This fraction contains nuclei in mid to late S-phase in addition to EdU-labeled nuclei that already entered into G2 phase with 4C DNA content.

In summary, the small size of molecules participating in the EdU labeling and detection allows for fast and efficient detection of the incorporated EdU without using harsh conditions, which may adversely affect the quality of data especially in plant flow cytometry. Simple and efficient EdU-based biparametric flow cytometry can therefore be readily incorporated into studies involving both monocot and dicot plant species and can be a very effective tool to assess culture health and proliferation status or in experiments based on plant cell cycle synchronization, stress treatments and genetic modifications.

### EdU analysis of S-phase cells in rice root meristems

**Figure 6:**
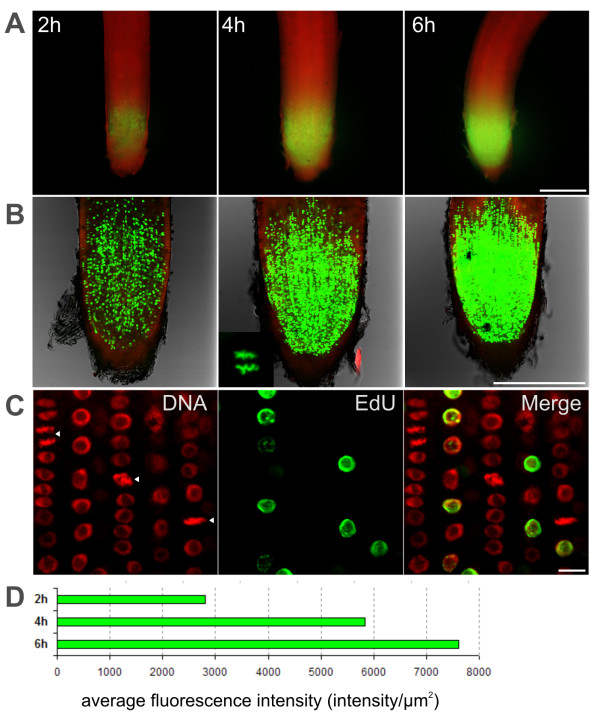
**Quantitative analysis of EdU incorporation on rice root meristems using EdU assay**. Rice roots (*O. sativa *L. ssp. japonica 'Nipponbare') were incubated for 2, 4, 6 hours with 20 μM EdU which was detected using Alexa Fluor 488 azide (green). **(A) **Fluorescence stereo microscopy images of EdU-labeled root tips. Transmission images of roots were pseudocolored red and merged with green EdU signal. Scalebar 300 μm. **(B) **Laser scanning confocal microscopy images of single optical sections of 6 μm (optical depth) on the median plane of rice root tips. Cell wall autofluorescence (red pseudocolor) and transmission images were overlaid onto green EdU signal. Inset photo shows a close-up of a 4 h EdU-treated cell with EdU-Alexa Fluor 488 labeled segregating chromosomes. Scalebar 300 μm. **(C) **Confocal images of EdU-labeled and DAPI-stained (DNA) cells from the meristem region of 2 h EdU treated root tips. Arrowheads (from left to right) indicate an anaphase, a prophase and a metaphase cell with well-preserved morphology. Scalebar 10 μm. **(D) **Quantitation of average EdU signal intensity of the meristem region after 2, 4 and 6 hours EdU incubation.

**Figure 7:**
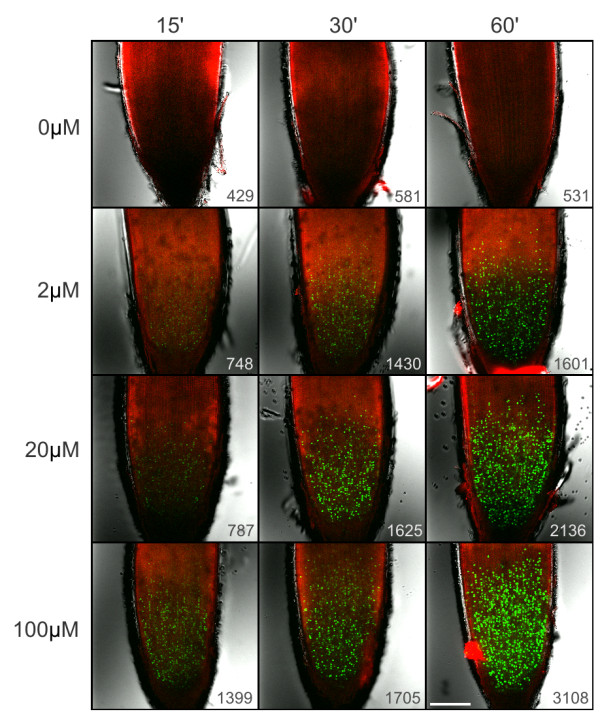
**The effect of EdU concentration and incubation time on rice root tip EdU assay**. EdU assay on rice (*O. sativa *L. ssp. japonica 'Nipponbare') root tips were shown for the indicated EdU concentrations and incubation durations. Control root tips (0 μM) were treated with 0.1% DMSO (vehicle) and incubated with Alexa488-containing assay cocktail to assess the level of nonspecific background and tissue autofluorescence in green channel. Cell wall autofluorescence (red pseudocolor) and transmission images were overlaid onto green EdU-Alexa Fluor 488 signal. Average green signal intensities (intensity/μm^2^) of the meristem regions were shown at the lower right corners. Higher detector sensitivity was used for green channel detection as compared to Figure 6B. Scalebar: 150 μm.

Immunolocalization of BrdU on thick plant tissues with dense cell layers is especially difficult due to cell wall and plasma membrane penetration problems of large (150 kDa) antibody molecules. To address these limitations, mechanical sectioning of plant material has been adopted [22]. However, sectioning protocol is tedious, time-consuming and requires expertise and specialized equipment. Moreover, reconstruction of the original three-dimensional tissue conformation from the individual sections is complicated. Click-reaction based EdU proliferation assay uses a small fluorochrome molecule (0.6 kDa) for detection [13]. Following successful application of EdU proliferation assay in various cultured cells with intact cell walls (Figure 2), we have tested EdU labeling on intact roots of germinating rice seedlings which have considerably thicker roots than, for example, Arabidopsis seedlings. Root tips of germinating rice were incubated for 2, 4 and 6 hours with 20 μM EdU then fixed with formaldehyde in a detergent-containing buffer and finally washed and incubated with EdU-detection reagent. EdU-labeled meristematic regions of roots were easily detectable with a fluorescence stereo microscope (Figure 6A). For detailed analysis of the meristem region and quantitation of the signal intensity, we have used confocal laser scanning microscope (Figure 6B). Single optical sections of 6 μm (optical depth) on the median plane of rice root tips were captured and average green fluorescence intensity of the 500 μm-long conical meristem area was measured and plotted (Figure 6D). Root contours, quiescent center and columella cells were identified by lignin autofluorescence of the cell walls [23]. No proliferation activity was detected in the quiescent center or in columella cells even after 6 hours of EdU incubation (Figure 6B). In the meristem region, however, EdU incubation time-dependent intensity increase was easily visible and quantifiable. Since EdU-detection protocol does not require acid or heat denaturation or digestion of DNA, chromosome morphology of the meristem region was also perfectly preserved. After DNA counterstaining, scoring of mitotic and EdU-labeling indices was also possible on individual optical sections with clearly defined mitotic phases (Figure 6C), however global measurement of average EdU incorporation in the meristem zone proved to be a faster and more convenient way for determination of proliferation activity in thick root tips of rice. Unlike elongation and differentiation zones of roots, the root meristematic zones display high proliferation activities with short cell cycle times. EdU labeling durations of 4 and 6 hours resulted in many EdU-labeled mitotic figures in root tips of rice. Inset photo of Figure 6B shows EdU-labeled anaphase chromosomes after 4 hours of EdU incubation. Presence of such post-metaphase figures also suggests that the incorporation of EdU into DNA does not cause chromosome segregation abnormalities during mitosis. Since EdU-labeled mitotic figures were detected very rarely after 2 h EdU incubation, we predict that the average G2 phase duration in rice root tip meristems (*O. sativa *L. ssp. japonica 'Nipponbare') should be between 2 and 4 hours. To determine the relationship between EdU concentration and labeling duration, rice root tips were treated with 0, 2, 20 and 100 μM EdU for 15, 30 and 60 minutes (Figure 7). Similar to the experiments with Arabidopsis suspension cultures (Figure 4), higher EdU concentrations allowed shorter EdU pulse durations. Comparable EdU labeling intensities at the root meristem regions were obtained with 15, 30 and 60 minutes of EdU incubations using 100, 20 and 2 μM EdU, respectively (Figure 7). Successful application of EdU assay on relatively thick rice root tip meristems suggests that the protocol can be applied to other plant organs such as leaves, seeds, shoot meristems, stems or flowers using the delivery methods already optimized for BrdU assays such as floating of seedlings [24], imbibition of seeds [25], liquid culturing of excised inflorescences [26] and direct submerging of intact leaves [27]. Practical and quantitative nature of root proliferation assay will find its application in various fields of plant science research such as developmental biology and stress physiology. Moreover, use of low magnification fluorescence stereo microscopy allows rapid screening of various experimental conditions therefore can be particularly suitable for high throughput screening studies.

## Conclusions

The EdU-based S-phase assay presented here affords a simple and rapid yet robust complement to previously validated methods of proliferation analysis in plant cells. Unlike antibody-based BrdU assay, EdU-based S-phase assay does not require cell wall digestion or DNA denaturation. This is not only advantageous from the point of morphological preservation of the samples, but also saves considerable amount of experimental time. EdU labeling and detection protocol can easily be adapted to various plant species regardless of cell wall thickness, composition or structure. The assay will be particularly useful in plant flow cytometry analyses due to superior preservation of isolated nuclei during the detection protocol. Apart from its application in cultured cells and isolated nuclei, the method is also well suited for quantitative proliferation analysis on thick tissues such as roots. Taking these data into account, we conclude that practical, versatile and quantitative nature of this robust S-phase assay will find its application in various fields of plant science research and will certainly be the new gold standard replacing BrdU-based immunolocalization assays.

## Methods

### Plant growth conditions

Plant suspension cultures were subcultured weekly. Culture medium and hormone concentrations are shown in Table 2. Rice (*O. sativa *ssp. japonica 'Nipponbare') seeds were surface sterilized for 5 min with 5% sodium hypochlorite solution (undiluted commercial bleach) then for 30 seconds with 70% ethanol solution. Seeds were washed 3 × 10 min with sterile distilled water and soaked in distilled water until germination started (24-36 h). Germinating seeds were transferred onto Petri dishes containing 1% agar in half strength MS medium (1/2 MS). Petri dishes were kept near vertically to promote unidirectional growth of root tips.

**Table 2:** Hormone concentrations and media used for plant cultures.

	Growth Medium	2,4-D (mg/L)	NAA (mg/L)	Kinetin (mg/L)
Alfalfa (*M. sativa *ssp. Varia A2)	MS	1	-	0.2

Arabidopsis (*A. thaliana *ecotype Columbia)	MS	-	0.5	0.05

Grape (*V. berlandieri *× *V. rupestris *cv. 'Richter 110')	MS	-	1	0.2

Maize (*Z. mays*, cv. H1233)	N6 M (LP40)	0.5	-	-

Rice (*O. sativa *ssp. japonica cv. 'Unggi 9'	G1	1	-	-

Tobacco (*N. tabacum *cv. Petit Havana SR1)	MS	-	1	0.2

2,4-D: 2,4-Dichlorophenoxyacetic acid. NAA: 1-Naphthaleneacetic acid. MS: Murashige and Skoog medium [29]. N6 M (LP40): Maize culture medium [30] with 40 mg/L L-proline. G1: Rice culture medium [31,32]

### Immunolocalization of BrdU

BrdU (5-bromo-2'-deoxyuridine, Sigma *catalog no: B5002*) stock solution was prepared as 30 mM aliquot in DMSO and kept in freezer. Two-days-old suspension culture of Arabidopsis was incubated for 2 hrs with 10 μM BrdU in its own culture medium. Cells were then fixed 15 min in 4% (w/v) formaldehyde solution in phosphate buffered saline (PBS) with 0.1% Triton X-100. Eight percent (2× concentrated) formaldehyde stock solution was prepared as following: Paraformaldehyde powder (Fluka *cat. no: 76240*) was dissolved in water by heating to about 60°C inside a fume hood and a drop of concentrated KOH was added as heating and alkaline pH depolymerizes paraformaldeyde. After cooling to room temperature, pH was set to neutral pH with dilute H_2_SO_4 _[5]. Aliquots of this stock fixer can be frozen for a few months. This 2× formaldehyde solution was then mixed 1:1 with 2× PBS (1× PBS contains 2.7 mM KCl, 1.47 mM KH_2_PO_4_, 137 mM NaCl, 8 mM Na_2_HPO_4_, pH7.4) and Triton X-100 was then added to a final concentration of 0.1% which provides uniform fixation with reduced cell shrinkage. Fixed cells were washed 2 × 5 min with PBS and 1 × 5 min with 0.5% MES (2-N-morpholinoethanesulfonic acid) pH 5.8. Cell walls were partially digested 30 min with chromatographically purified lyophilized enzymes from Worthington Biochemical Corporation (Lakewood, NJ, USA). The enzyme mixture was 1% cellulase (*Cat no: LS02601*) and 0.5% pectinase (*Cat. no: LS04297*) in 0.5% MES, pH 5.8. After washing with PBS (3 × 5 min), cells were settled on poly-L-lysine coated multiwell slides, excess solution was removed and cells were permeabilized 30 min with 0.5% Triton X-100 in PBS to allow antibody penetration. Following 3 × 5 min washes with PBS containing 5 mM MgSO_4_, cells were incubated 30 min in 20 units/ml chromatographically purified, ribonuclease- and protease-free DNase I (Worthington Biochem. Corp. *cat. no: LS006331*) in PBS with 5 mM MgSO_4_. Fifty times concentrated stock solution of DNase I (1000 units/ml) was prepared by dissolving 2500 units of lyophilized powder in 1.25 ml 50% glycerol with 1 mM CaCl_2 _and kept in freezer in aliquots. DNase I solution was removed with 3 × 5 min washes with antibody buffer. Antibody buffer, (PBS^+^) contains PBS with 5% (v/v) fish gelatin (Sigma *cat. no: G7765*, to prevent nonspecific binding of antibodies) and 0.02% (w/v) sodium azide (Fluka *cat. no: 71290*, to inhibit bacterial growth during antibody incubations. For EdU assay sodium azide should not be used before click reaction). Cells were incubated 3 h at 37°C with monoclonal (clone BU-33) mouse anti-bromodeoxyuridine antibody (Sigma *cat. no: B8434*) diluted 1:200 in PBS^+^. Following 5 × 5 min washes with PBS^+^, cells were incubated 1 h at 37°C with rabbit anti-mouse Alexa Fluor 488 conjugated antibody (Invitrogen *cat no: A11059*) diluted 1:300 in PBS^+^. Cells were then washed 3 × 5 min with PBS containing 100 ng/ml DNA staining dye DAPI (4',6-diamidino-2-phenylindole, Invitrogen cat *no: D1306*) and mounted with Fluoromount-G anti-fade mounting solution (Southern Biotech, *cat no*: 0100-01).

### EdU-based proliferation assay

Two-days-old monocot and dicot plant suspension cultures were incubated 2 hrs with 10 μM EdU (Invitrogen *cat no: A10044*) in their own culture medium. Arabidopsis cultures (36 h-old) were incubated with various concentrations and durations as shown in Figure 4. Cells were then fixed 15 min in 4% (w/v) formaldehyde solution in phosphate buffered saline (PBS) with 0.1% Triton X-100. Addition of the detergent Triton X-100 in the fixer prevents cell shrinkage and it also partially permeabilizes the plasma membranes for small detection reagents of EdU assay. Moreover, quick penetration of the fixer allows better preservation of mitotic chromosomes. However, for experiments where preservation of cytoskeleton or cytoplasmic organelles is important, detergent should be omitted from the fixer and plasma membranes should be permeabilized after fixation as in BrdU immunolocalization protocol. Following 3 × 5 min PBS washes, 20-30 μl packed cell volume of cells were directly incubated 30 min at room temperature (RT) in EdU detection cocktail (Invitrogen, Click-iT EdU Alexa Fluor 488 HCS assay, *cat no: A10027*). For 1 sample reaction, following amounts of the kit components are mixed in 144 μl distilled water: 1.6 μl buffer additive (component F, kept frozen in small aliquots), 14 μl reaction buffer (Component D), 6.7 μl Copper (II) sulfate solution (Component E, 100 mM CuSO_4_) and 0.07 μl Alexa Fluor 488 azide (Component B, in 70 μl DMSO). Many azide-labeled fluorochromes other than Alexa Fluor 488 are available throughout the visible spectrum for multicolor labeling purposes. Click reaction requires Cu (I) which can be formed using CuSO_4 _in the presence of a reducing agent such as sodium ascorbate [28]. For experiments where defined buffer components are necessary, we have found that the use of detection cocktail with the following composition resulted in positive EdU labeling on Arabidopsis suspension cultures and on isolated rice and alfalfa nuclei: 4 mM CuSO_4_, 40 mM sodium ascorbate, 20 μM Alexa Fluor 488 azide in PBS (for intact cells) or in nuclei isolation buffer (for nuclei). To prevent oxidation of Cu (I) to non-catalytic Cu (II) species, the detection cocktail should be prepared freshly. Although the click reaction is not light sensitive, fluorochrome-containing solutions should not be exposed to strong light. After 2 × 5 min washes with PBS containing 100 ng/ml DAPI, an aliquot of cells were mounted in PBS. Glycerol-based (or high osmolarity) mounting mediums caused shrinkage during mounting; therefore PBS mounting is used for all cell lines. For root tip labeling, root tips of germinating rice seedlings (*O. sativa *L. ssp. japonica 'Nipponbare') were submerged into 20 μM (Figure 6) or 0, 2, 20, 100 μM EdU (Figure 7) in half strength MS medium. Root tips were then cut in detergent-containing fixer (see above) and fixed for 30 min at RT. Fixer was washed with PBS (3 × 10 min) and root tips were incubated in EdU detection cocktail (see above) for 30 min followed by PBS or PBS-DAPI washes (see above). Fluoromount-G anti-fade solution was used for mounting of root tips.

### Confocal laser scanning and fluorescence stereo microscopy

Confocal laser scanning microscopy was performed using Olympus Fluoview FV1000 confocal laser scanning microscope (Olympus Life Science Europa GmbH, Hamburg, Germany). Microscope configuration was the following: objective lenses: UPLSAPO 20× (dry, NA:0.75), UPLFLN 40× (oil, NA:1.3) and UPLSAPO 60× (oil, NA:1.35); sampling speed: 4 μs/pixel; line averaging: 2x; scanning mode: sequential unidirectional; excitation: 405 nm (DAPI and cell wall lignin autofluorescence) and 488 nm (Alexa Fluor 488); laser transmissivity: less than 1% and 5% were used for DAPI and Alexa Fluor 488, respectively; main dichroic beamsplitter: DM405/488; intermediate dichroic beamsplitter: SDM 490; DAPI and cell wall autofluorescence were detected between 425-475 nm and Alexa Fluor 488 was detected between 500-600 nm and pseudocolored red and green, respectively. Differential interference contrast (DIC) images were captured with 488 nm laser line. For rice root tip imaging and EdU-signal quantitation, single optical sections of 6 μm (optical depth) on the median plane of rice root tips were captured with 20× objective. Identical laser power and detection settings were used for quantitative analyses. For imaging of all root tips of Figure 7, a higher detector sensitivity setting (as compared to Figure 6) was used due to very short EdU pulse durations. The center of the root with the widest girth was determined by lignin autofluorescence signal of the cell walls. For quantitation of the green EdU signal, meristem regions were manually traced up to 500 μm distance starting from the quiescent center. Area measure tool of Olympus Fluoview software (version 1.7.2.2) was used for measurement of area and intensities at the root tips. Total intensity of the green signal (named as "integration" by Olympus Fluoview software) was divided by the measured meristem area to determine average intensity per square microns and plotted using Microsoft Office Excel 2003 software. For nuclear counterstaining and close-up view of chromosomes, DAPI (200 ng/ml) was used on EdU labeled (2 hrs) roots and observed by 40× objective. For fluorescence stereo microscopy, Olympus SZX12 stereo microscope was used. Alexa Fluor 488 images were captured with the "green" filter set: Excitation: 460-490 nm, dichroic beam splitter 505 nm, emission: 510-550 nm. Transmission photos were pseudocolored red and merged with fluorescence images. Composite images were prepared using "import image sequence" and "make montage" functions of ImageJ software (National Institutes of Health, USA, version 1.41).

### Nuclei isolation and Flow cytometry

Three-days-old rice and alfalfa cultures were incubated with 10 μM EdU for 8 hrs and 4 hrs, respectively. For unfixed nuclei preparations, 4d-old rice cells were 10 μM EdU-treated for 4 hrs. Three-days-old Arabidopsis cultures were incubated either with 0.1% DMSO (as control) or with 10 μM EdU in DMSO for 15' and 30'. EdU-labeled and 0.1% DMSO-treated control cultures were chopped with a sharp razor blade in nuclei isolation buffer (45 mM MgCl_2_, 20 mM MOPS, 30 mM sodium citrate, 0.1% Triton X-100, pH 7.0) in 6 cm plastic Petri dishes on ice [17]. Nuclei (in 2 ml buffer) were filtered into 15 ml conical bottom tubes through 20 μm sieves and fixed on ice for 30 min by the addition of 8% formaldehyde solution (see above) to a final concentration of 1%. Fixed nuclei were washed twice with 2 ml 0.01% Triton X-100 containing PBS by centrifugation at 4°C (10 min, 400 g/1500 rpm) using Heraeus Labofuge 400R (Thermo Fisher Scientific, Rockford, IL, USA) desktop centrifuge with swing-out rotor. Washed nuclei were incubated in 500 μl EdU-detection cocktail for 30 min at room temperature. Unfixed nuclei were centrifuged (10') and resuspended in 500 μl EdU-detection cocktail and incubated 30' at room temperature (Water and buffer component D in the cocktail recipe were replaced by nuclei isolation buffer for unfixed nuclei). After one wash (5') with PBS containing 0.01% Triton X-100 (for fixed nuclei) or with nuclei isolation buffer (for unfixed nuclei), nuclei were counterstained either with 100 ng/ml DAPI (for microscopy check) or with 5 μg/ml PI (propidium iodide, Invitrogen *cat. no: P1304 MP*) and analyzed on a FACSCalibur flow cytometer (Becton, Dickinson and Company, NJ, USA) with CellQuest software. Two fluorescence detectors are used with the standard 488 nm laser. Alexa Fluor 488-EdU intensity was detected between 515-545 nm (FL1 channel). For detection of PI intensity (DNA content) 564-606 nm emission range was used at FL2 channel. Side scatter versus forward scatter diagrams were used to locate and gate nuclear populations by particle size. Dot-plot diagram of "total PI fluorescence of a particle at FL2 channel" (FL2-A) versus "transit time of a particle at FL2 channel" (FL2-W) was used as secondary gating to exclude particles which are not fluorescent with PI staining. To locate the boundary of EdU-Alexa Fluor 488-labeled nuclei in biparametric plots (EdU threshold value) counts versus FL1-H (Alexa488-EdU channel, log scale) histograms were used. The left (major) peak of this histogram (with low green channel intensity) represents EdU unlabeled G1 and G2 populations while the higher green intensity second peak represents EdU-labeled nuclei. The right border of the leftmost major peak (where the unlabeled G1/G2 counts reach zero value) is selected as the EdU threshold value. EdU threshold values of control samples were determined by corresponding EdU-treated samples. The midpoint PI intensity (~300 PI intensity units) is selected as the vertical separating line for 2C DNA and 4C DNA contents in quadrant analyses.

## Competing interests

The authors declare that they have no competing interests.

## Authors' contributions

EK and FA performed the experimental work. DD and GVH acquired funding, provided plant materials and revised the manuscript. FA designed and coordinated the project. All authors read and approved the final manuscript.
